# Editorial: Physiology and Pathophysiology of Heat Shock Protein 60

**DOI:** 10.3389/fmolb.2020.604476

**Published:** 2020-10-30

**Authors:** Antonella Marino Gammazza, Celeste Caruso Bavisotto, Alberto J. L. Macario

**Affiliations:** ^1^Section of Human Anatomy, Department of Biomedicine, Neuroscience and Advanced Diagnostics (BiND), University of Palermo, Palermo, Italy; ^2^Euro-Mediterranean Institute of Science and Technology (IEMEST), Palermo, Italy; ^3^Department of Microbiology and Immunology, School of Medicine, University of Maryland at Baltimore-Institute of Marine and Environmental Technology (IMET), Baltimore, MD, United States

**Keywords:** Hsp60, genetic chaperonopathies, acquired chaperonopathies, post translational modifications, hepatocellular carcinoma, cardiovascular disease, chaperonotherapy

The authors were invited to participate in this Research Topic before the COVID-19 pandemic outbreak and we all were very enthusiastic at the prospect of assembling a series of articles on the physiological and pathological roles of the chaperone Hsp60. In the introductory overview, we stated that Hsp60 is a Group I chaperonin and key component of the chaperoning system, typically residing inside mitochondria but also occurring in other cellular compartments and extracellularly. Noteworthy is the variety of locales beyond the mitochondrion in which Hsp60 occurs such as the cytosol, plasma-cell membrane, intercellular space, body fluids (blood, lymph, cerebrospinal fluid), and secretions (saliva, urine). This diversity of residences suggests many functions for Hsp60 in the body physiology and predicts widespread consequences if it malfunctions, affecting a variety of tissues and organs. Thus, Hsp60 is one of the components of the chaperoning system that is of interest in Medicine across a wide range of specialties. Therefore, we thought that by bringing together a series of articles on Hsp60 in physiology and physiopathology we would provide those in the biomedical sciences with a resource useful in research and practice. Unfortunately, SARS-CoV-2 burst onto the stage and forced many potential contributors to direct their efforts toward the pandemic and its consequences in all aspects of academic life rather than write an article for our Research Topic. Fortunately, however, a few scientists could still send their manuscripts, and these constitute the substance of this Editorial as present in the following paragraphs.

Please, note that all Figures and Tables cited in this Editorial can be found in the article discussed.

de Lima Filho et al. present an interesting work reporting a correlation between of anti-Hsp60 antibodies and cardiovascular disease in bedridden elderly patients. Frailty in elderly people represents a clinical challenge because it affects almost every organ and entails a general susceptibility to infectious agents and stressors of all kinds. de Lima Filho et al. studied 57 elderly bedridden subjects whose clinical and anthropometric characteristics are displayed in Table 1 of their article. They assessed the Framingham Risk Score of these subjects in relation to the levels of Hsp60 antibody in their blood (this score is a gender specific algorithm that is applied to estimate the future cardiovascular risk of a patient over a period of 10 years). It can be seen in Table 3 that there is a positive correlation between the Framingham Score and the serum levels of Hsp60 antibodies. The authors suggest that immune activation resulting in the production of anti-Hsp60 antibodies is associated with cardiovascular risk in bedridden patients with aging fragility. This observation raises the interesting question of how Hsp60, which is typically an intracellular protein, gets in contact with the immune system. Is the chaperonin released into the blood as cells die and disintegrate and/or is it secreted by an active mechanism unleashed by accumulation of the chaperonin inside stressed cells? Whatever the mechanism, anti-Hsp60 antibodies serve as a biomarker potentially useful for following up bedridden patients with ageing fragility.

The contribution by Duan et al. deals with the participation of Hsp60 in the biology of the cardiovascular system be it normal development and physiology or pathophysiology, namely in the mechanism of some cardiovascular diseases. As we stated in the introductory paragraph of this Editorial, Hsp60 is present in virtually all tissues and organs and participates in a wide range of physiological and pathophysiological phenomena. Illustrative examples of Hsp60 roles in the cardiovascular system are discussed in the article by Duan et al. They focus on Hsp60 roles in the initiation and progression of heart failure and in the mechanism of atherosclerosis, both nicely illustrated in their Figures 1 and 2, respectively. But before dealing with the pathologies, the authors explain the roles of Hsp60 in cardiomyocytes, vascular endothelial cells and vascular smooth muscle cells, providing the basic elements to understand the mechanisms of Hsp60 in the pathogenesis of cardiovascular diseases. These and various other parts of the contribution by Duan et al. are useful sources of information on the diverse roles of Hsp60 in the maintenance of mitochondrial integrity with its implications in the physiology of the heart muscle and vascular smooth muscle, and in inflammation and apoptosis pertaining to atherosclerosis and heart disease.

The possible involvement of Hsp60 in hepatocellular carcinoma (HCC) is discussed by Hoter et al. HCC is a frequent and serious tumor against which there is no efficacious treatment. Therefore, new studies and new approaches for treatment must be carried out and tested. In this context, Hsp60 is an attractive target for research because it will provide information useful for disease diagnosis and for developing treatment strategies centered on the chaperonin. Hoter et al. discuss the role of Hsp60 in carcinogenesis and apoptosis, which can be anti- or pro and, therefore, the issue is controversial. Data in the literature are varied and various interpretations have been proposed, so the general picture is at times confusing as illustrated by the information provided in Table 1. Interestingly, the authors discuss the role of Hsp60 in carcinogenesis of HCC by analyzing the role of the chaperonin in comorbidities that favor HCC such as viral infections (hepatitis B and hepatitis C viruses), alcoholism, aflatoxins, and steatohepatitis. For those cases in which Hsp60 is suspected of having a carcinogenic effect the use of chaperonin inhibitors (negative chaperonotherapy) is discussed and a list of inhibitors is provided in Table 2.

Since there is only one Hsp60 gene in the human genome, it is puzzling to see that the chaperonin displays so many roles in a variety of intra- and extra-cellular locales. One wonders how one molecule can perform such a variety of tasks. One factor may be subtle structural changes associated with post-translational modifications (PTM) that generate a range of slightly different molecules able to interact with various other molecules in their surroundings, a different set of interactors for each PTM and locale. This topic is discussed in the article by Caruso Bavisotto et al., in which a comprehensive review of the known PTMs of Hsp60 is critically presented. The various functions of Hsp60 are displayed in Table 1 and illustrative examples of PTMs are shown in Table 2. Some modifications such as nitration, ubiquitination, S-nitrosylation, and S-guanylation are depicted in Figure 1, and these and many others are mapped on the linear Hsp60 amino acid sequence in Figure 2. These graphic materials should make useful teaching tools. A discussion is also presented on the possible role of PTMs in making Hsp60 pathogenic, when this chaperonin causes a chaperonopathy, for there are a range of diseases in which Hsp60 plays a pathogenic role, either primary or secondary. Clinical identification of these Hsp60 chaperonopathies is key to optimal patient management because these conditions are amenable to treatment with agents directed to the chaperonin that can inhibit or eliminate the malfunctioning chaperonin -negative chaperonotherapy-, as mentioned in the article on Hsp60 and hepatocellular carcinoma by Hoter et al. in this Research Topic.

Hsp60 chaperonopathies, discussed by Rodriguez et al., can be genetic or acquired with the former being the result of a mutation in the gene encoding the chaperonin. In the acquired chaperonopathies, the Hsp60 gene is normal but its product is not, or at least it does not function normally and causes a disease or contributes as an accessory cause of a disease. Examples of acquired Hsp60 chaperonopathies are various inflammatory and autoimmune conditions and several types of cancers (see e.g., the articles by Hoter et al. and by Dulan et al. in this Research Topic). While acquired chaperonopathies are widespread and with high incidence, genetic Hsp60 chaperonopathies are not. Two have been studied extensively, in which the human Hsp60 gene (designated *HSPD1*) bears the mutations D29G or V98I (also known as D3G and V72I if the first 26 amino acids that form the mitochondrial targeting sequence are excluded), causing MitCHAP-60 disease and spastic paraplegia 13 (SPG13, respectively. These are discussed by Rodriguez et al. in their article, in which detailed accounts of the complex structure of Hsp60 in its functional multimolecular arrangement and of its chaperoning cycle are provided for the benefit of the reader. MitCHAP-60 is an autosomal-recessive condition characterized by hypomyelinating leukodystrophy and SPG13 is an autosomal dominant form of hereditary spastic paraplegia. In both chaperonopathies, the effect of the mutation is manifested by a weakness of the multimolecular associations that are necessary for Hsp60 to function correctly. Formation of these associations by the mutant chaperonin is not as effective as the wild type counterpart, and the functional molecular teams and networks are destabilized and tend to fall apart. Since Hsp60 is typically a mitochondrial chaperone, its pathogenic mutations affect mostly mitochondrial function, which has a devasting impact on the nervous system, explaining why the associated disorders although affecting various tissues are predominantly neurological diseases.

## Author Contributions

All authors listed have made a substantial, direct and intellectual contribution to the work, and approved it for publication.

## Conflict of Interest

The authors declare that the research was conducted in the absence of any commercial or financial relationships that could be construed as a potential conflict of interest.

